# Tissue Engineering Bone Using Autologous Progenitor Cells in the Peritoneum

**DOI:** 10.1371/journal.pone.0093514

**Published:** 2014-03-28

**Authors:** Jinhui Shen, Ashwin Nair, Ramesh Saxena, Cheng Cheng Zhang, Joseph Borrelli, Liping Tang

**Affiliations:** 1 Department of Bioengineering, University of Texas at Arlington, Arlington, Texas, United States of America; 2 Division of Nephrology, University of Texas Southwestern Medical Center at Dallas, Dallas, Texas, United States of America; 3 Departments of Physiology and Developmental Biology, University of Texas Southwestern Medical Center at Dallas, Dallas, Texas, United States of America; 4 Texas Health Physicians Group, Texas Health Arlington Memorial Hospital, Arlington, Texas, United States of America; 5 Department of Biomedical Science and Environmental Biology, Kaohsiung Medical University, Kaohsiung, Taiwan; University of Wisconsin-Madison, United States of America

## Abstract

Despite intensive research efforts, there remains a need for novel methods to improve the ossification of scaffolds for bone tissue engineering. Based on a common phenomenon and known pathological conditions of peritoneal membrane ossification following peritoneal dialysis, we have explored the possibility of regenerating ossified tissue in the peritoneum. Interestingly, in addition to inflammatory cells, we discovered a large number of multipotent mesenchymal stem cells (MSCs) in the peritoneal lavage fluid from mice with peritoneal catheter implants. The osteogenic potential of these peritoneal progenitor cells was demonstrated by their ability to easily infiltrate decalcified bone implants, produce osteocalcin and form mineralized bone in 8 weeks. Additionally, when poly(l-lactic acid) scaffolds loaded with bone morphogenetic protein-2 (a known osteogenic differentiation agent) were implanted into the peritoneum, signs of osteogenesis were seen within 8 weeks of implantation. The results of this investigation support the concept that scaffolds containing BMP-2 can stimulate the formation of bone in the peritoneum via directed autologous stem and progenitor cell responses.

## Introduction

Bone loss often occurs as a result of open fractures, osteomyelitis, fractures which fail to heal, congenital malformations, tumors, and in a more general sense, osteoporosis. In recent years, significant progress has been made in the development of tissue engineered bone designed to replace or bridge large bone defects [1]–[3]. Typical tissue engineering strategies involve the use of an implant, usually in the form of a 3-dimensional scaffold, which integrates with existing bone tissue to restore bone and to some extent, the function of the damaged bone [4]–[6]. In recent years, adult mesenchymal stem cells (MSCs) have shown great promise in regenerating tissue engineered tissues and organs [7], [8]. However, these approaches are still plagued by limitations associated with the recovery, differentiation ability and survival of autologous MSCs [9]–[13]. Therefore, there remains a need for better means to generate functional tissue by tissue engineering techniques, including bone.

Ossification of the peritoneum is a pathological condition often associated with patients undergoing peritoneal dialysis (PD) or as a result of traumatic splenic rupture or peritonitis [14]–[16]. Although inflammatory responses are believed to contribute to peritoneal ossification [15], the process(es) governing peritonitis-mediated ossification is not clear. Recent studies carried out in our laboratories have found that biomaterial-mediated inflammatory responses can prompt the recruitment of MSCs with multipotency, including osteogenic potential/activities [17]. In addition, we have recently discovered that the implantation of peritoneal catheters prompts the immigration of MSCs to the peritoneal cavities in humans (unpublished results). Based on these observations, we assume that, with the localized release of an osteogenic agent, the recruited MSCs would differentiate into osteoblasts to promote mineralization of tissue engineered bone scaffolds *in situ*.

To test this hypothesis, we first analyzed the compositions and osteogenic properties of progenitor cells in lavage fluids of animals following intraperitoneal implantation of biomaterials. Using decalcified bone matrix as an osteoconductive material, we assessed the osteogenic activity of these cells in the peritoneum. Finally, to explore the applicability of this process for *in situ* bone tissue engineering, we developed and used poly l-lactic- acid (PLLA) scaffolds, combined with bone morphogenetic protein-2 (BMP-2), and evaluated the potential for stimulating the production of viable bone in the peritoneum by inducing osteogenic reactions from autologous progenitor cells.

## Materials and Methods

### Ethics Statement

The animal use protocols (A11-008, A07-030) were reviewed and approved by the Institutional Animal Care and Use Committee of the University of Texas at Arlington.

### Materials

Goat anti-mouse SCF and Nanog antibodies and rabbit anti-mouse antibodies were obtained from Santa Cruz Biotechnology Inc. (Santa Cruz, CA). Anti-mouse CD45, Sca-1, c-kit, CD34, FLK2-, CD3, B220, TER-119-, antibodies (rat anti mouse) to various stem cell markers CD105, CD29 and CD44, along with secondary antibody streptavidin PE/Cy5.5, and donkey anti-rat –APC were obtained from eBioscience (San Diego, CA, USA). Rat anti mouse CD105 was obtained from Santa Cruz Biotechnology (Dallas, TX, USA). Biotin conjugated lineage antibody cocktail was obtained from Miltenyi Biotec (Miltenyi Biotec Inc, Auburn, CA). Bone morphogenetic protein-2 (BMP-2) was obtained from R&D Systems (R&D Systems, Minneapolis, MN). PLLA was obtained from Medisorb 100L 1A (Lakeshore Biomaterials, AL, USA) with inherent viscosity of 1.9 dL/g.

### Methods

#### Mouse peritoneal cell collections

Balb/c mice (4∼6 months) were used in this experiment. Mice were implanted with polyurethane umbilical vessel catheter (2 cm length, 5.0 FR, Sentry Medical Products, Lombard, IL, USA) based on modified procedure published earlier [18], [19]. Briefly, the mice were sedated with Isoflurane inhalation. Following sterilization with 70% ethanol, a small incision (∼5 mm) was made and two sections of catheter were implanted in the peritoneal cavities. The incisions were then closed with stainless steel wound clips. After implantation, the mice were euthanized at different times (0 h, 6 h, 12 h, 18 h, 1 d, 2 d, 4 d, 7 d, 10 d, 14 d) with carbon dioxide inhalation. The peritoneal cells were then recovered via peritoneal lavage with 5 ml of sterile saline twice. The isolated cells were then characterized by determining the expression of various cell markers and via cell differentiation studies.

#### Flow cytometry analyses and cell differentiation of peritoneal progenitor cells

For flow cytometry analysis, RBC lysing buffer (Sigma Chemical Co., St Louis, MO) was used to remove red blood cells from each sample following the manufacturer's instructions as previously described [20]. The cell density was adjusted to 5×10^6^/ml and then stained with monoclonal antibodies including anti-mouse CD105, CD29, CD45, CD44, CD3, B220, Mac-1, TER-119, or biotin conjugated lineage antibody cocktail (CD3, B220, CD11b, CD14, Ter 119, Miltenyi Biotec), Streptavidin secondary antibody, Sca-1, c-kit, CD34, FLK2. Lin^−^Sca-1^+^Kit^+^CD34^−^FLK2- are widely used markers for long term hematopoietic stem cells (HSCs) [20], [21], while CD105^+^CD29^+^CD44^+^CD45^−^ is well recognized as the marker set for MSCs [22], [23]. Stained cells were analyzed on BD FACSCalibur (BD Bioscience, San Jose, USA) to determine the types and percentages of peritoneal cells. Osteogenic differentiation of peritoneal progenitor cells was performed on confluent cells in the presence of recombinant BMP-2 (R&D Systems, Minneapolis, MN) for 3 weeks as previously described [24]. Calcium-rich deposits by osteoblasts were then evaluated using Alizarin Red S staining [24]. Adipogenic, neurogenic and myogenic differentiation of peritoneal progenitor cells was performed and analyzed similar to earlier publications [17].

#### Induced bone formation in peritoneum

Both decalcified bone collagen scaffolds and porous PLLA scaffolds were used for triggering bone formation in the peritoneum. It is well established that decalcified bone scaffolds contain necessary bone morphogenetic protein for inducing bone formation [25], [26]. Decalcified femur bone scaffolds (∼1.5 mm×1 mm×15 mm in size) were prepared according to published procedures [27], [28]. It is estimated that there are 3 mg of BMP-2 per gram of demineralized dentin [29], and ∼50–100 ng of a combination of BMPs per 25 mg of bovine bone matrix [30]. PLLA scaffolds were fabricated following salt leaching technique [31]. PLLA scaffolds have been widely used in bone tissue engineering research [32], [33]. For this study, PLLA scaffolds were fabricated based on optimal scaffold design criteria related to pore size and structure, as established previously [34]–[37]. To stimulate localized osteogenic differentiation, ethanol sterilized PLLA scaffolds (5 mm×5 mm×5 mm in size with a pore size of 150 to 300 µm) were immersed in osteogenic differentiation solution (complete medium supplemented with 50 µg/ml ascorbic acid-2-phosphate, 10 nM dexamethasone, 7 mM β-glycerolphosphate, and 1 µg/ml BMP-2) overnight, then lyophilized prior to implantation. BMP-2 is a known osteogenic differentiation agent and a few studies have shown high levels of osteogenic activities in various stem cells at 10–50 ng/ml [38], [39]. Our pilot studies have shown that the *in vivo* release rates are approximately 5% (∼50 ng) per scaffold per day for a period of 2 weeks. For *in vivo* testing, the scaffolds, including untreated scaffolds as control, were implanted in the peritoneal cavities [18], [19]. The implants were isolated at 16 hours, 2, 6 and 12 weeks for histological evaluation.

#### Histological Evaluation of peritoneal implants

All explants were frozen, cryosectioned, fixed, and used for various histological evaluations as described earlier [17], [40]. H&E stain was used for providing a general overview of tissue structures. Immunohistochemical analyses were performed for assessing the presence of stem and progenitor cell markers including; SCF and Nanog, and osteoblast markers - osteocalcin and alkaline phosphatase - based on previous studies [40]. Alkaline phosphatase activity (AP activity) was tested using a biochemical assay obtained from Sigma (St. Louis, MO, USA). Calcium content change was tested by Alizarin Red S staining and von Kossa staining. All stained sections were observed under light microscopy (Leica DM LB) and the images analyzed using ImageJ, as described earlier [17], [40].

#### Statistical analyses

The extent of cell recruitment, stem cell marker expression and mineralization were analyzed using One way ANOVA. PLLA scaffold mineralization at the end of 8 weeks was evaluated using Student's t-test. The statistical significance was determined at p<0.05.

## Results

### Recruitment and characterization of peritoneal fluid cells following dialysis in mice

**Figure 1:**
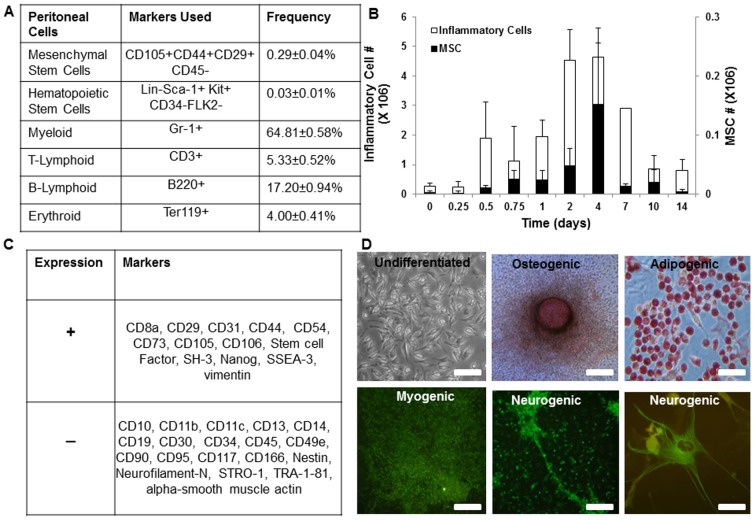
Multipotent MSCs exist in Balb/c mice peritoneal effluents. (A) Peritoneal cell population from animals with 2 day implants. (B) Recruitment of inflammatory cells and MSCs in mice peritoneal cavity after catheter implantation. (C) Expression of surface markers on peritoneal MSCs. (D) Peritoneal cells differentiated into specific lineages appropriate conditions. Morphology of undifferentiated cells is compared with cells differentiating into specific lineages like osteogenic (Alizarin Red S stain), adipogenic (Oil Red O stain), myogenic (α-smooth muscle actin) and neurogenic (NF-M and Class III β-tubulin stain). (Mag 200×, scale bar 100 µm, statistical significance of cell numbers at various time points tested using One Way ANOVA, *p<0.05).

A murine peritoneal implantation model was used to study the feasibility of using the peritoneum as a site for *in vivo* tissue regeneration. For that, it was essential to first investigate the cell populations that exist/arrive in the mice peritoneum following introduction of an implant. Polyurethane umbilical vessel catheters (2 cm in length) were implanted into the mouse (n = 5) peritoneal cavity to mimic the trauma and foreign body response caused by peritoneal dialysis procedures. After catheter implantation, peritoneal lavage fluid was collected at various time points up to day 14 from both catheter-implanted and control animals. Not surprisingly, after implantation for 2 days, we predominantly observed a number of inflammatory cells like T-cells (5.33±0.52%), B-cells (17.20±0.94%), myeloid cells (64.81±0.58%) and erythroid cells (4±0.41%) (Figure 1A). Interestingly, we also found that there were two unique sub-populations in the effluent cells that shared markers identical to those of MSCs (CD105^+^CD44^+^CD29^+^CD45^−^) and HSCs (Lin^−^Sca-1^+^Kit^+^CD34^−^FLK2^−^) in nearly each of the animals, these cells accounted for 0.29±0.04% and 0.03±0.01% respectively (Figure 1A–B). Interestingly, peritoneal MSCs also express many progenitor cell markers, including CD8a, CD29, CD31, CD44, CD54, CD73, CD105, CD106, Stem Cell Factor (SCF), SH-3, Nanog, SSEA-3, and vimentin (Figure 1C). They also stained negative for CD10, CD11b, CD11c, CD13, CD14, CD19, CD30, CD34, CD45, CD49e, CD90, CD95, CD117, CD166, Nestin, Neurofilament-N, STRO-1, TRA-1-81, or alpha-smooth muscle actin. Similar cells were also identified from the peritoneal effluents of human patients with End Stage Renal Disease (ESRD) (unpublished observation). To test the functionality and plasticity of these peritoneal progenitor cells, we assessed their multipotency by culturing them *in vitro* in the presence of various differentiation-inducting media. Specifically, the undifferentiated cells exhibited fibroblast like morphology; the culture of these cells in specific differentiation mediums led to differentiation into osteogenic, adipogenic, neurogenic and myogenic phenotypes (Figure 1D). Collectively, these results suggest that the implantation of catheters in the peritoneum of adult mice prompted the migration of multipotent MSCs and other progenitor cells that express markers similar to those expressed on bone marrow stem cells into the area.

### Calcification of decalcified bone implants

**Figure 2:**
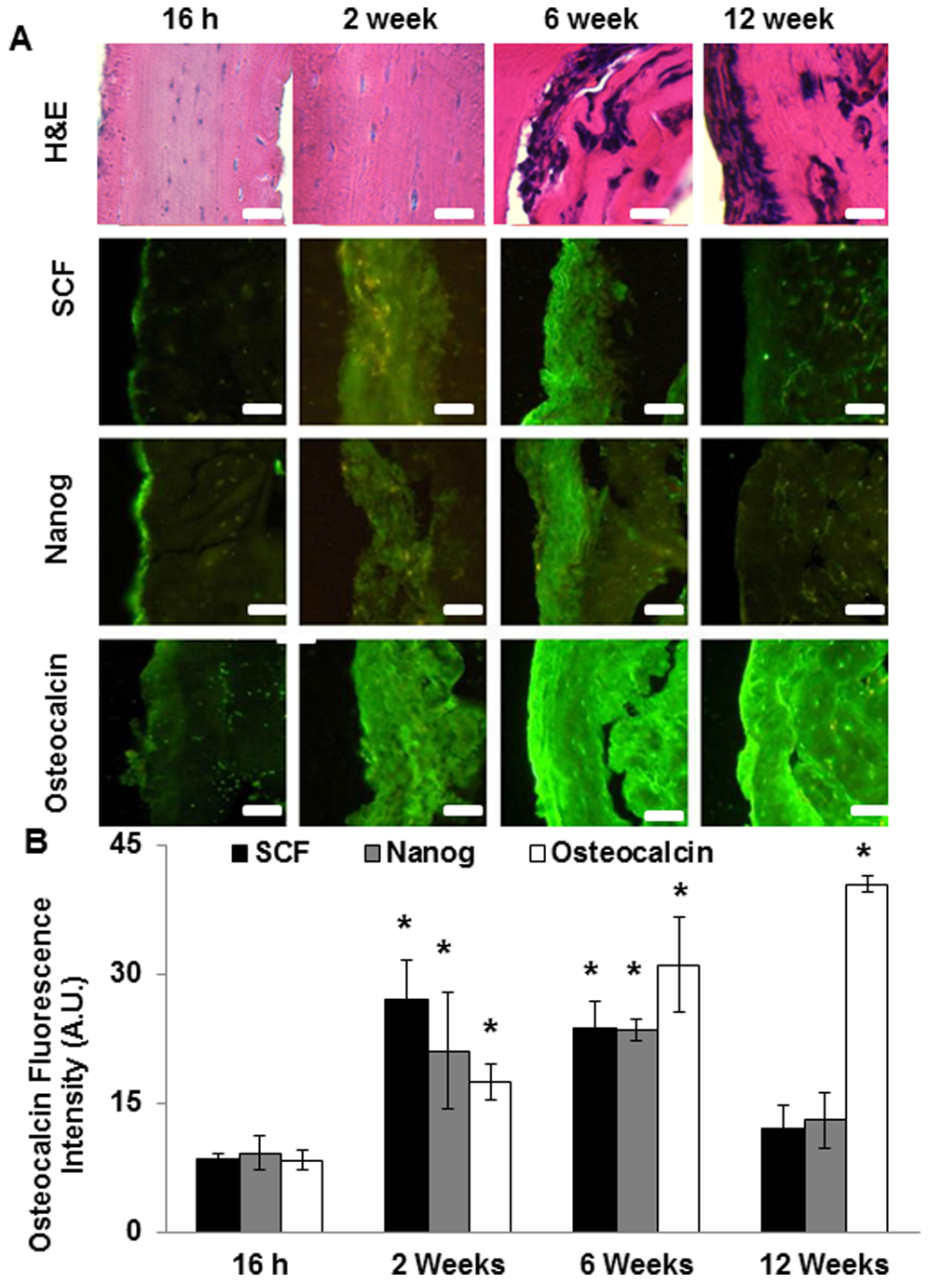
Progenitor cells are found around decalcified peritoneal bone implants. (A) Hematoxylin & Eosin (H&E) staining and immunohistochemical staining of SCF, Nanog and Osteocalcin on decalcified bone scaffold 16 hours, 2 weeks, 6 weeks and 12 weeks after implantation, positive stained as green. (B) Quantification of SCF, Nanog and osteocalcin expression was performed using NIH ImageJ. (Mag 200×, scale bar 200 µm, statistical significance of cell surface marker expression at various time points tested using One Way ANOVA, * p<0.05).

To determine the ability of these MSCs cells to form bone tissue in the peritoneum, we initially used decalcified bone scaffolds (approximately 1.5 mm×1 mm×15 mm in size). By doing so, we were able to study the ability of intraperitoneally implant-recruited MSCs to differentiate into bone forming cells and to produce mineralized bone tissue. Since peritoneal MSCs were found to express SCF and Nanog, both markers were used to assess the extent of MSC recruitment following scaffold implantation. H&E staining of the bone scaffolds showed an increase in eosinic staining from 16 hours to 12 weeks along with high cell infiltration by 12 weeks (Figure 2A). Interestingly, shortly after implantation (16 hour), there were many recruited cells including SCF+ and Nanog+ progenitor cells and an increased number on the surfaces of scaffold implants (Figure 2A). As expected, implant-associated osteoblast activity remained low as reflected by slight osteocalcin production on the tissue: scaffold interface of all the evaluated time points. After two weeks, SCF+/Nanog+ progenitor cells (stained in green) were found to be almost 2 times that found at 16 hours, indicating the recruitment and infiltration of progenitor cells into bone scaffolds (Figure 2B). The numbers of SCF+/Nanog+ cells remained the same through week 6 and were substantially reduced in number by week 12. By week 2, osteocalcin expression increased almost 2 times compared to that at the end of 16 hours. By 6 weeks this increase was almost 3 times, and by week 12 osteocalcin expression was almost 4 times that at 16 hours. Interestingly, over the 6 to 12 week period as the levels of osteocalcin increased, the expression of SCF and Nanog returned to the same levels as that at the end of 16 hours, suggesting the differentiation of the SCF+/Nanog+ progenitor cells into osteocalcin+ osteoblasts.

### Mineralization in decalcified bone implants

**Figure 3:**
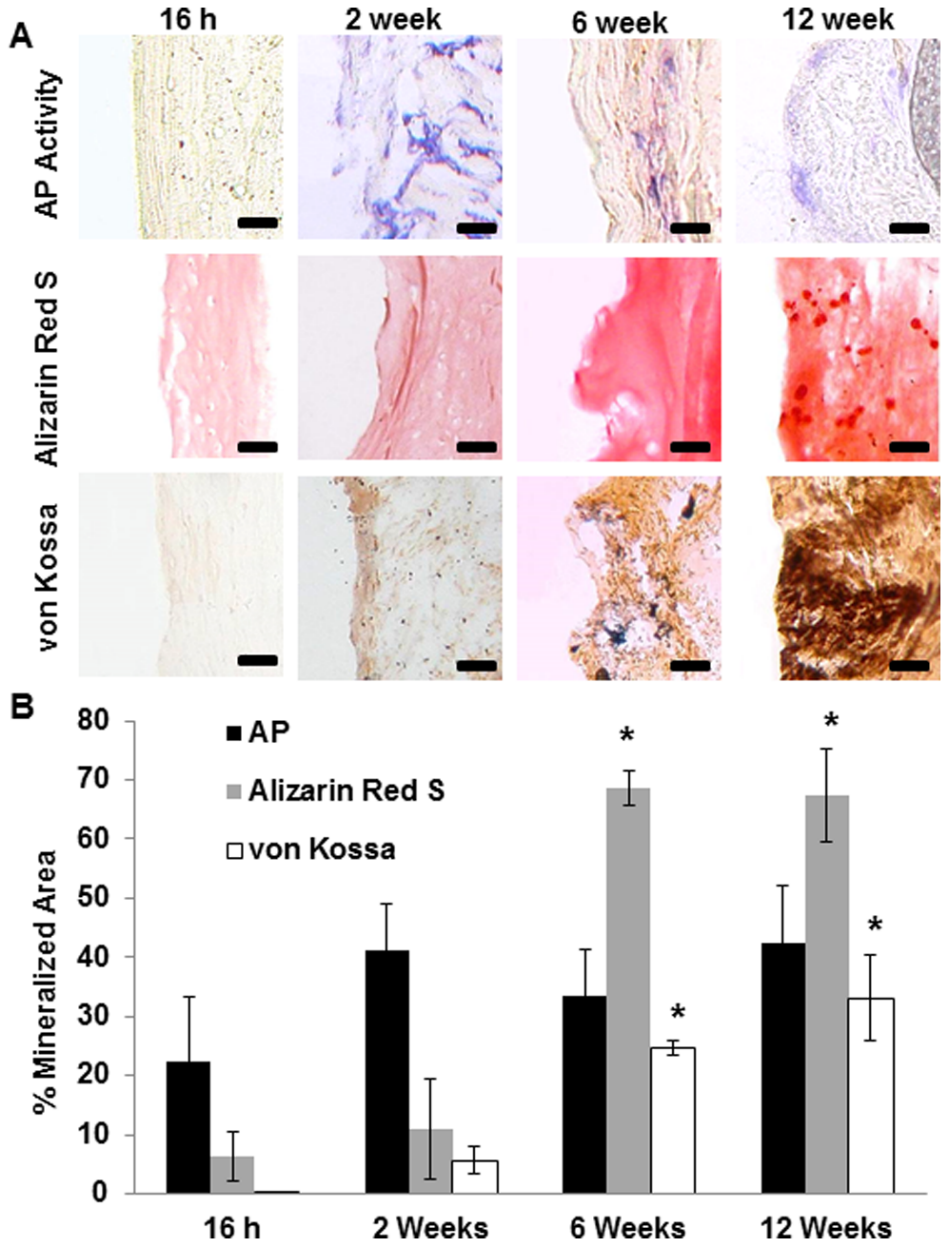
Mineralization of decalcified bone implants. Alkaline phosphatase (AP) assay and calcification staining of implanted decalcified bone implants at 16 hours, 2 weeks, 6 weeks, and 12 weeks (A). Quantification of AP, and calcification based on Alizarin Red S and von Kossa staining was performed using NIH ImageJ (B) (Mag 200×, scale bar 200 µm, statistical significance of mineralization at various time points tested using One Way ANOVA, * p<0.05).

Mineralization of the decalcified bone scaffolds in the peritoneum was determined based on alkaline phosphatase (AP) activity, Alizarin Red S staining and von Kossa staining (Figure 3A). The area fraction of the implants that were mineralized was determined using ImageJ (Figure 3B). We found that AP activity increased at week 2 and that the tissue associated AP activity remained stable from weeks 2 to 12. Alizarin Red S stain (orange or red) and von Kossa stain (brown or black deposits) were also carried out to assess scaffold mineralization. There was no significant increase in calcium content between 16 hours to 2 weeks. However, scaffold mineralization dramatically increased during the period between week 6 and 12 by more than 7 fold compared to the 16 hours to 2 week period.

### Ossification of scaffolds implanted within the peritoneum

**Figure 4:**
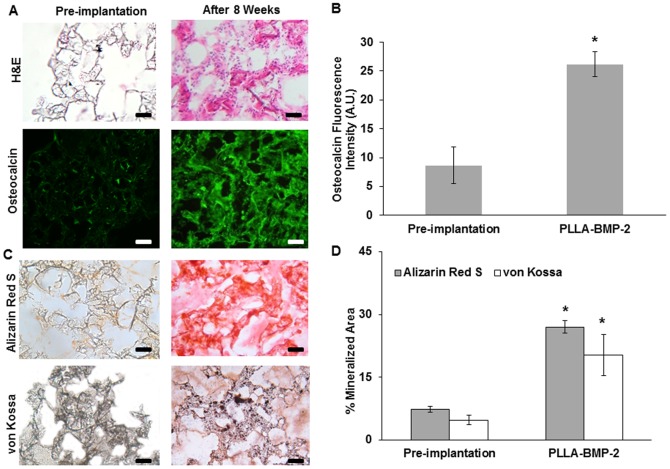
Mineralization and pro-osteogenic activity in PLLA-BMP2 scaffold implants in Balb/c mice peritoneal cavity after 8 weeks. (A) H&E staining and osteocalcin staining of PLLA-BMP2 scaffold after 8 weeks. (B) Quantification of osteocalcin intensity was performed using NIH ImageJ. (C) Examination of calcium deposits on scaffold based on Alizarin Red S and von Kossa staining. (D) Quantification of calcified area was performed using NIH ImageJ. (Mag 200×, scale bar 200 µm, significance of scaffold implant after vs. before implantation using Student's t-test, *p<0.05).

Use of decalcified bone scaffolds to induce osteogenic differentiation of stem and progenitor cells in the peritoneum is not a clinically relevant approach. To find an alternative bone tissue engineering strategy, subsequent studies were carried out using poly-l- lactic acid (PLLA) polymer scaffolds that were loaded with BMP-2, which is an osteogenic differentiation agent and has been used clinically for bone tissue engineering [41], [42]. Interestingly, even without stem cell pre-seeding, PLLA scaffolds promote the recruitment of MSCs (∼0.35±0.10%) by day 3. By week 8, we found substantial infiltration of cells into the scaffolds (as seen by the H&E staining). Coincidentally, many infiltrated cells expressed osteocalcin, indicating osteoblast activity (Figure 4A). There was a nearly 5 fold increase in osteocalcin expression (Figure 4B). Signs of mineralization in the form of calcium and phosphate deposits were observed within the scaffold as well (Figure 4C). Quantification of these markers of osteogenic activity showed a 5 fold increase in mineralization at the end of 8 weeks in PLLA scaffolds loaded with osteogenic differentiation agent BMP-2 (Figure 4D).

## Discussion

For years, peritoneum has been used for evaluating host responses to biomaterials that have subsequently been employed in human subjects to meet clinical needs [18], [43]–[49]. Recently, a few studies have even explored the possibility of using the peritoneum to grow visceral organs like bladder, uterus and vas deferens [50], [51]. Most of these organ/tissue regeneration strategies involve the transplantation of cell-seeded scaffolds. Additional studies attempted to grow blood vessels by implanting biomaterials into the peritoneum [52], [53]. These studies showed that free-floating implants within the peritoneum acquired layers of macrophage derived myofibroblasts and collagen matrix along with mesothelial cells and undifferentiated cells bearing markers similar to those expressed on bone marrow stem cells [52], [53]. Although several recent works, including ours, indicated the presence of progenitor cells in the peritoneum [53]–[55], the potential of using peritoneal stem cells for tissue engineering application has not been explored. Since tissue calcification has been observed following peritoneal dialysis, and splenic rupture [18], [44], [46]–[48], [56], it is possible that pathogenic processes might lead to recruitment and osteogenic differentiation of progenitor cells in the peritoneum. To support these observations, we found that progenitor cells expressing various MSC surface markers were recruited to peritoneal implants with their numbers increasing dramatically until day 4, post implantation. Based on the expression of cell surface markers and lineage specific differentiation, we found that the peritoneal stem cells were similar to bone marrow stem cells [17], [57]. Such cells were multipotent, as they had the ability to differentiate into osteogenic, adipogenic, neurogenic and myogenic phenotypes. The mechanism or process for stem and progenitor cell recruitment to the peritoneal cavity following biomaterial implantation has yet to be determined. It is likely that foreign body reaction-associated inflammatory signals are responsible, since anti-inflammatory agents have been shown to reduce stem cell recruitment to the subcutaneous implants [17]. In addition, inflammatory chemokines, such as CCL2, CCL3, CCL4 and CXCL12 have been shown to promote mesenchymal stem cell migration [58]–[63].

Osteogenic differentiation is essential for stem cell-mediated bone regeneration [64]. To promote osteogenic differentiation of peritoneal stem cells, we first used decalcified bone scaffolds and then BMP-2-loaded PLLA scaffolds. It is well established that decalcified bone scaffolds contain osteogenic agents essential for osteogenic differentiation [65], [66]. We found the presence of SCF+/Nanog+ progenitor cells reached their peak within 2 weeks of implantation and the cell density remained stable for up to 6 weeks, post implant. However, after week 6, the reduction of SCF+/Nanog+ progenitor cell number coincided with an increase of osteoblast osteocalcin activities and bone mineralization. A recent study has reported elevated expression of Nanog during undifferentiated state of stem cells followed by a drop in the levels upon osteoblastic differentiation [67]. Our results support that scaffold-containing osteogenic agents prompted the differentiation of progenitor cells into osteoblasts.

The formation of mineralized bone tissue in the peritoneum is a unique phenomenon that may be related to a disease - peritoneal ossification (PO). The cause of PO is not fully understood. However, it is generally believed that chronic inflammatory responses associated with peritoneal dialysis may be responsible [68]–[70]. It is possible that mineralized scaffolds and PO share a similar mechanism [16]. In fact, a few studies have suggested that an inflammatory stimulus, as that associated with implantation in the peritoneum, could lead to osteogenic cell migration from surrounding bone into the peritoneum or the inflammatory stimulus acts on stem cells to produce mesoblasts and osteoblasts [69], [71], [72].

While this phenomenon opens up a new vista in tissue regenerative strategies, there are additional opportunities for further advancement. For example, better designed scaffolds which can release bioactive chemokines at a more desirable rate could possibly enhance this bone formation phenomenon. We have made further advancement in scaffold development and investigated bone regeneration in critical sized defect models [2], [64]. The results from this study indicates the suitability of the peritoneal environment for autologous progenitor cell recruitment and differentiation into bone. These findings can pave the way for future research by exploring smarter materials to facilitate the development of more efficient strategies that engineer bone *in vivo* in the peritoneum so it can be readily transplanted to the defective sites, such as critical size defects, as needed.
